# Azole Use in Agriculture, Horticulture, and Wood Preservation – Is It Indispensable?

**DOI:** 10.3389/fcimb.2021.730297

**Published:** 2021-09-07

**Authors:** Lise Nistrup Jørgensen, Thies Marten Heick

**Affiliations:** Department of Agroecology, Aarhus University, Slagelse, Denmark

**Keywords:** fungicide resistance, plant pathogens, yield losses, human health, azole market

## Abstract

Plant pathogens cause significant damage to plant products, compromising both quantities and quality. Even though many elements of agricultural practices are an integral part of reducing disease attacks, modern agriculture is still highly reliant on fungicides to guarantee high yields and product quality. The azoles, 14-alpha demethylase inhibitors, have been the fungicide class used most widely to control fungal plant diseases for more than four decades. More than 25 different azoles have been developed for the control of plant diseases in crops and the group has a world market value share of 20-25%. Azoles have proven to provide long-lasting control of many target plant pathogens and are categorized to have moderate risk for developing fungicide resistance. Field performances against many fungal pathogens have correspondingly been stable or only moderately reduced over time. Hence azoles are still, to date, considered the backbone in many control strategies and widely used as solo fungicides or as mixing partners with other fungicide groups, broadening the control spectrum as well as minimizing the overall risk of resistance development. This review describes the historic perspective of azoles, their market shares and importance for production of major crops like cereals, rice, oilseed rape, sugar beet, banana, citrus, and soybeans. In addition, information regarding use in amenity grass, in the wood preservation industry and as plant growth regulators are described. At the end of the review azoles are discussed in a wider context including future threats following stricter requirements for registration and potential impact on human health.

## Introduction

Plant pathogens reduce the yield and quality of agricultural products and may cause substantial economic losses. The latter affect food security at household, national and global levels (Savary et al., 2019). The reporting of yield losses varies significantly depending on region and target crops; it is, thus, difficult to obtain reliable data to estimate the overall impact of plant pathogens. Two independent studies concluded that crop losses for five of the commodities most widely grown: Wheat, rice, maize, potatoes and soybean, were in the range of 20 to 30 % (Oerke, 2006; Savary et al., 2019).

Food security and ‘Zero Hunger’ are among the global goals declared by the United Nations (Anon, 2015). Efforts to reduce fungal diseases are crucial for guaranteeing sufficient high-quality food for a growing world population. Modern agriculture has been increasingly reliant on the application of fungicides to mitigate fungi-related crop losses and fulfill this goal. Sterol biosynthesis inhibitors have been the predominant fungicide class controlling fungal diseases and securing yield for many decades. Within this group, DeMethylation Inhibitors (DMI; FRAC group G1) have proven to be particularly efficacious against a broad range of fungal pathogens. In the following, we will use the terms ‘azole’ and ‘DMI’ synonymously when we address this group of fungicides. The development of systemic fungicides has led to a change in the paradigm for control because their systemic movement in the plant created a threshold-oriented fungicide application using various risk models in support of optimizing timing (Russell, 2005). Following the introduction of benzimidazoles in the 1960s, the arrival of DMI fungicides can be seen as the second major wave of systemic fungicides.

The target of DMIs is the 14α-demethylase enzyme (CYP51) in the ergosterol biosynthetic pathway of fungal pathogens of both plants and humans (Price et al., 2015). Ergosterol is a predominant sterol in many fungal cell membranes in higher fungi, such as Ascomycetes, Basidiomycetes, but not in Oomycetes. Binding to the CYP51 enzyme leads to an accumulation of eburicol in the cell and, consequently, the production of ergosterol is stopped (Siegel, 1981). The qualitative changes of sterol composition impact the plasma membranes containing high levels of sterols, leading to altered membrane fluidity. The result is a delay in fungal growth or, in higher concentrations, a fungicidal effect (Buchenhauer, 1987; Scheinpflug, 1987).

The first compounds belonging to the demethylase inhibitors were derivatives of piperidine, pyridine, pyrimidine and morpholine (Russell, 2005). However, the group of DMIs dominating the fungicide market are imidazoles and triazoles. The list of active ingredients belonging to the azole group is long and reflects the fact that a development took place in all major chemical companies present at the time (**Table 1**). Despite great similarities among the different azoles, every single compound has its own specific activity spectrum on targeted plant pathogens; some azoles are used mainly as a seed treatment, others only as foliar products, and some are effective against both seed-borne and foliar diseases. Apart from their fungicidal effect, certain azoles also share a growth-regulating property. Furthermore, azoles are used as wood preservation agents.

**Table 1:** List of azoles development and timeline and main uses (modified from Russel, 2005).

Common azole	Year of introduction	Chemical group	Company	Description of application areas
Imazalil	1973	Imidazoles	Janssen	Fruit, including citrus, apples, pears, bananas; cucumbers; roses; cereal seed treatments
Prochloraz	1977	Imidazoles	Boots	Fruit; field crops; mushrooms; turf; avocados; mangoes; cereals.; oilseed rape
Fenarimol	1975	Pyrimidines	Dow	Fruit, including bananas, cherry, grapes, pears; ornamental plants and trees; turf
Nuarimol	1975	Pyrimidines	Eli Lilly	Fruit, including pome and stone; grapevines; cucurbits; cereals
Prothioconazole	2002	Triazolinthiones	Bayer	Cereals; oilseed rape; specialist in fusarium control
Pyrifenox	1986	Pyridines	Maag	Fruit, including apples, pears, cherries, peaches, apricots; sugar beet; vegetables
Triforine	1968	Piperazines	Celamerck	Cereals; cucurbits; mushrooms; almonds; fruit, including blueberries, cherries, peaches, apples, grapes, mangoes; hops; ornamentals, including roses
Bitertanol	1979	Triazoles	Bayer	Fruit, including apples, bananas; cereals; peanuts; ornamentals and flowers; beans; soybeans; wood preservative
Bromuconazole	1990	Triazoles	Rhone Poulenc	Cereals; fruit; vegetables; grapevine; turf; ornamentals
Cyproconazole	1986	Triazoles	Sandoz	Cereals; vegetables, including peas, beans, and asparagus; oilseed rape; sugar beet; fruit, including apples, peach; almonds
Difenoconazole	1988	Triazoles	Ciba	Vegetables, including carrots, asparagus, brassicas; potatoes; cereals; sweet corn; cotton; oilseed rape
Diniconazole	1993	Triazoles	Sumitomo	Peanuts; grapes; ornamentals, including roses; cereals
Epoxiconazole	1990	Triazoles	BASF	Cereals; sugar beet; corn
Fenbuconazole	1988	Triazoles	Novartis	Cereals; grapevines; top fruit; stone fruit; bananas; pecans; rice
Fluquinconazole	1992	Triazoles	Schering	Cereals – take-all seed treatments
Flusilazole	1983	Triazoles	Dupont	Fruit, including apples, pears, apricots, plums, peaches, bananas, grapes; sugar beet; oilseed rape; cereals
Flutriafol	1983	Triazoles	ICI/Nikon	Cereals, including corn; soybeans; apples
Hexaconazole	1986	Triazoles	Zeneca	Grapes; apples; pears; bananas; vegetables; some small grain cereals; wood preservative
Mefentrifluconazole	2020	Triazoles	BASF	Cereals; turf; applications for use in other crops sent in for registration
Metconazole	1992	Triazoles	Kureha/Cyanamid	Cereals; oilseed rape; fruit, including blueberry, cherry, gooseberry, nectarine, peaches, plum; pistachio; turf and sod
Myclobutanil	1986	Triazoles	Rohm & Haas	Perennial and annual crops; turf; landscape ornamentals; fruit trees; grapes
Penconazole	1983	Triazoles	Ciba	Grapes; fruit, including apples, pears, peaches, plums, apricots, strawberries; ornamentals; hops; vegetables, including cucumbers and tomatoes
Propiconazole	1979	Triazoles	Janssen	Cereals; maize; wild rice; peanuts; almonds; sorghum; oats; pecans; fruit, including apricots, plums, prunes, peaches, and nectarines; wood preservation
Tebuconazole	1986	Triazoles	Bayer	Cereals; grapes; peanuts; vegetables, including onions, peas, pepper; bananas; sugarcane; wood preservation
Tetraconazole	1988	Triazoles	Bayer	Sugar beet; wheat; grapes; apples
Triadimefon	1969	Triazoles	Bayer	Cereals; peas; grapes; cucurbits; sugarcane
Triadimenol	1973	Triazoles	Bayer	Cereals; beet crops; brassicas; grapes
Triticonazole	1992	Triazoles	Bayer	Cereals; turf; ornamentals
Paclobutrazole	2006	Triazoles	Syngenta	Plant growth regulator in ornamentals; mangoes; cotton; corn; chili; grapes

In addition to their contribution to modern agriculture, fungicides are currently controversially discussed and face several challenges. The use of azoles in agriculture is highly debated in light of significant regulatory restrictions issues related to, for example, endocrine disruption, environmental fate relating to persistence and metabolites, fungicide resistance issues and particularly cross-resistance to azoles used in human or animal medicine for control of fungi infections. This paper underlines the importance of azoles in major crops, including cereals (*Triticum aestivum*, *Hordeum vulgare*), oilseed rape (*Brassica napus*), sugar beet (*Beta vulgaris*), bananas (*Musa* spp.), rice (*Oryza sativa*: Asian rice or *Oryza glaberrima*: African rice), soybean (*Glycine max*), oranges (*Citrus* spp.) and turfgrass mainly on golf courses. The paper also includes information on the use of azoles applied as growth regulators and in the wood preservation industry. Furthermore, the main constraints that azoles are currently facing are discussed.

## History of Azoles

The use of azoles for non-medical purposes has a wide span from agriculture and horticulture to the prevention of post-harvest losses. Azoles target a broad spectrum of fungal pathogens in various crops (**Table 2**). As one of the first azoles, imazalil was registered in the late 1970s as a seed treatment widely used in cereals and the protection of seed potatoes (Russell, 2005). It has also been used widely as a post-harvest treatment for citrus, pome fruits and bananas, including the control of storage diseases, such as *Penicillium* in citrus production. Several other azoles entered the market (e.g. triadimefon/triadimenol or propiconazole) during the late 1970s and early 1980s in terms of leaf disease control in cereal and fruit production (Russell, 2005). In the early 1980s, prochloraz was introduced and rapidly adopted in Europe to control eyespot on cereals (*Oculimacula yallundae*), which was a major challenge at that time because of the buildup of fungicide resistance to benzimidazole (Griffin and King, 1985). Over time, the first azoles introduced have been replaced to a large extent by other active ingredients, such as tebuconazole (1992), difenoconazole (1994), epoxiconazole (1994), bromuconazole (2000) and, from 2002, prothioconazole. These newer azoles have been extensively used to control seed-borne, leaf and ear blight diseases. After some time with no new active azoles being registered, mefentrifluconazole focusing on the control of Septoria tritici blotch (STB, *Zymoseptoria tritici*), was launched in 2020 and it is also expected to control major target diseases in other crop segments (Strobel et al., 2020; Ishii et al., 2021).

**Table 2:** List of examples of main target diseases for which DMIs are applied.

Main crops	Examples of main target diseases for DMIs
Wheat (*Triticum aestivum*)	*Puccinia* spp., *Blumeria graminis*, *Zymoseptoria tritici*, *Fusarium* spp., *Pyrenophora tritici repentis*, *Oculimacula* spp.
Barley (*Hordeum vulgare*)	*Puccinia* spp.*, Blumeria graminis, Pyrenophora teres, Rhynchosporium commune*
Rye (*Secale cereale*)	*Puccinia* spp.*, Blumeria graminis*, *Rhynchosporium secale*
Oat (*Avena sativa*)	*Puccinia corona, Blumeria graminis, Pyrenophora avenae*
Maize (*Zea mays*)	*Kabatiella zeae*, *Fusarium* spp., *Setosphaeria turcica*, *Colletotrichum graminicola, Puccinia polysora*
Rice (*Oryza sativa*)	*Pyricularia oryzae*, *Rhizoctonia solani*, *Cochliobolus miyabeanus*, *Villosiclava virens*
Oilseed rape (*Brassica napus*)	*Sclerotinia sclerotiorum*, *Alternaria* spp.*, Pyrenopeziza brassicae*, *Plenodomus lingam*, (also used for growth regulation)
Sugar beet (*Beta vulgaris*)	*Cercospora beticola, Erysiphe betae, Uromyces betae, Ramularia beticola*
Sugarcane (*Saccharum officinarum*)	*Bipolaris sacchari, Puccinia kuehnii, Sporisorium scitaminies *
Potato (*Solanum tuberosum*)	*Alternaria* spp.
Orange (*Citrus sinensis*)	*Penicillium digitatum, Penicillium italicum, Elsinor fawcettii, Alternaria* spp.
Banana (*Musa ssp*.)	*Pseudocercospora fijiensis, Fusarium oxysporum, Pseudocercospora musae, Colletotrichum musae*
Soybean (*Glycine max*)	*Phakopsora pachyrhizi, Cercospora kikuchii.Corynespora cassiicola*
Apple (*Malus domestica*)	*Venturia inaequalis, Monilia digitata*
Wine (*Vitis vinifera*)	*Erysiphe necator*
Golf courses	*Microdochium nivale, Rizoctonia solani, Colletothrichum cereale, Clarirededia jacksonil, Magnaporthiopsis poae*
Pot plants	*Erysiphe* spp., *Puccinia* spp., (also used for growth regulation)

## Azoles Market Share

**Figure 1:**
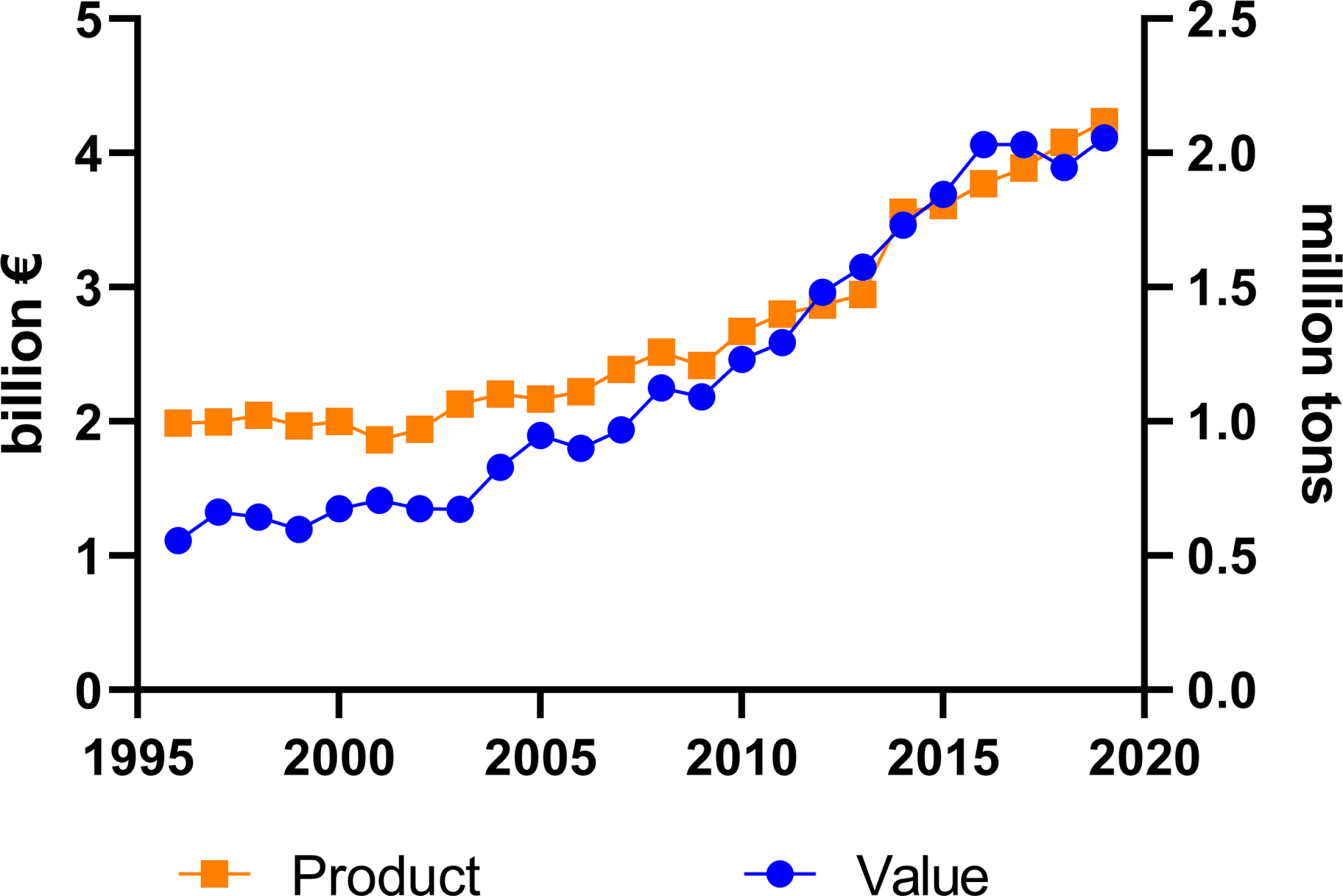
Amounts of agricultural azoles product (tons) and their market value (€) measured during 24 years (source: Bayer Internal Source).

**Figure 2:**
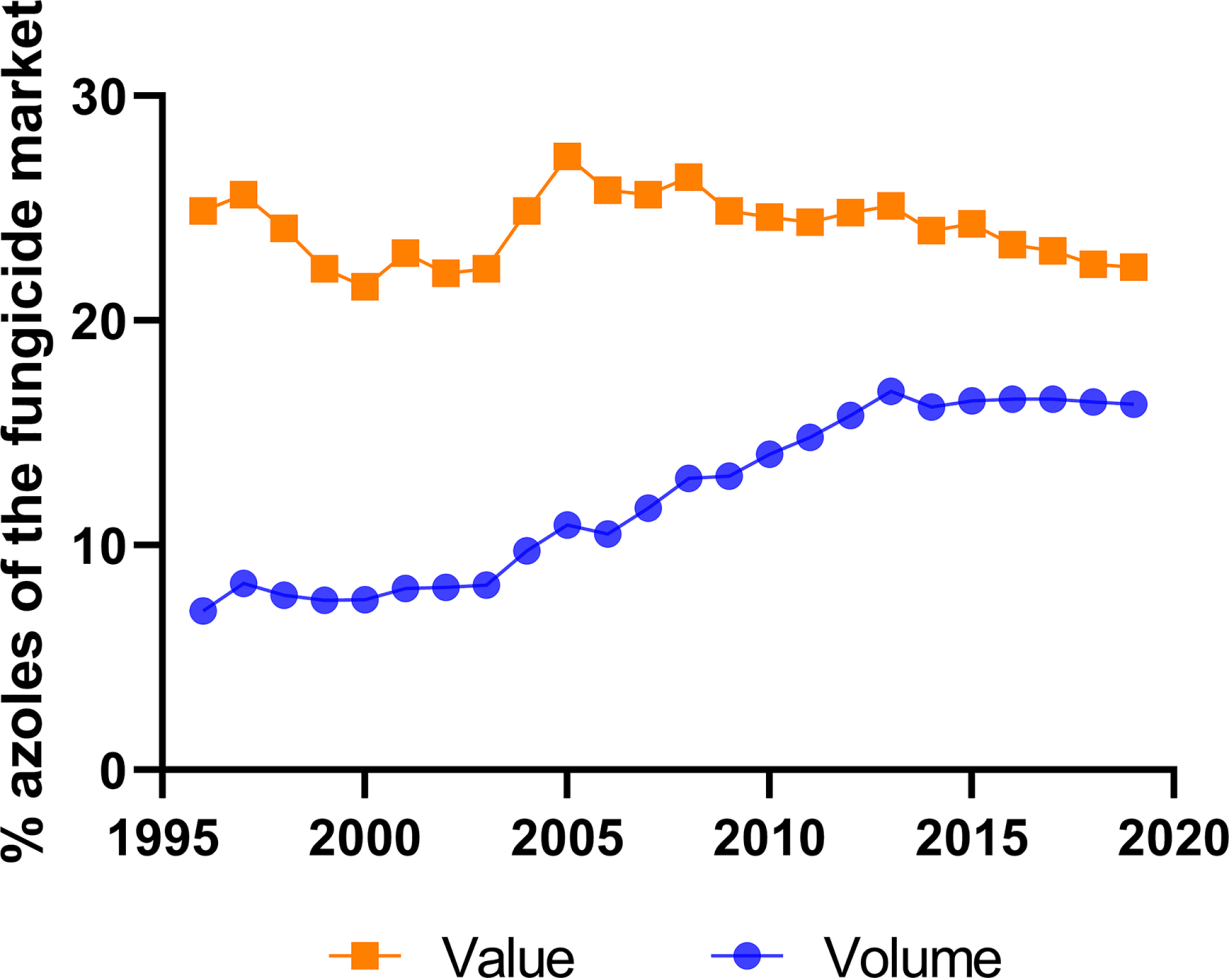
Importance of azoles in the overall global fungicide market. Azoles share (%) of global fungicide marked shown in value and volume during 24 years (source: Bayer Internal Source).

**Figure 3:**
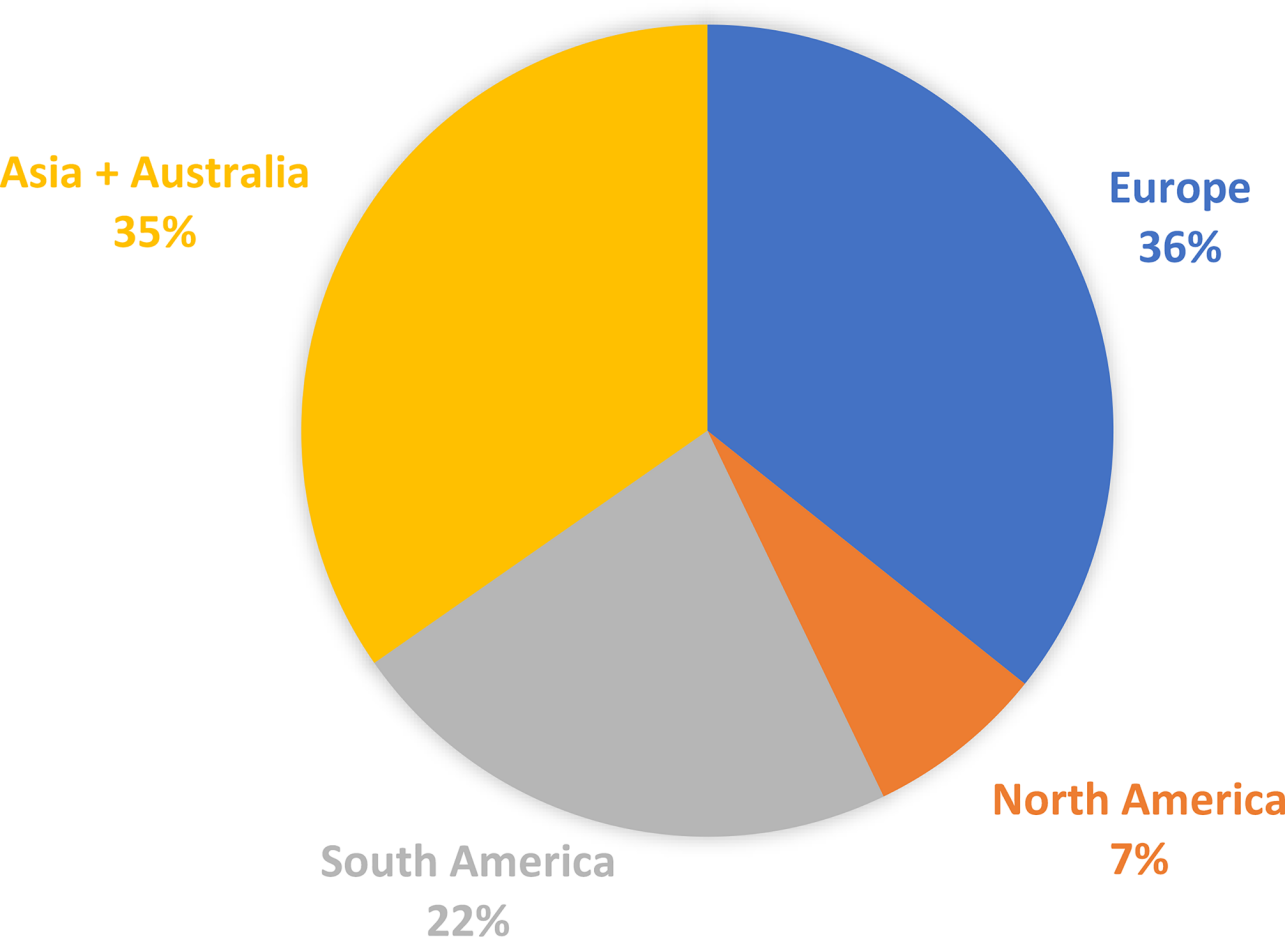
Distribution of azoles volumens sold in agriculture in the major continents. Data from 2018 (source AgbioInvestor/Kynetec).

**Figure 4:**
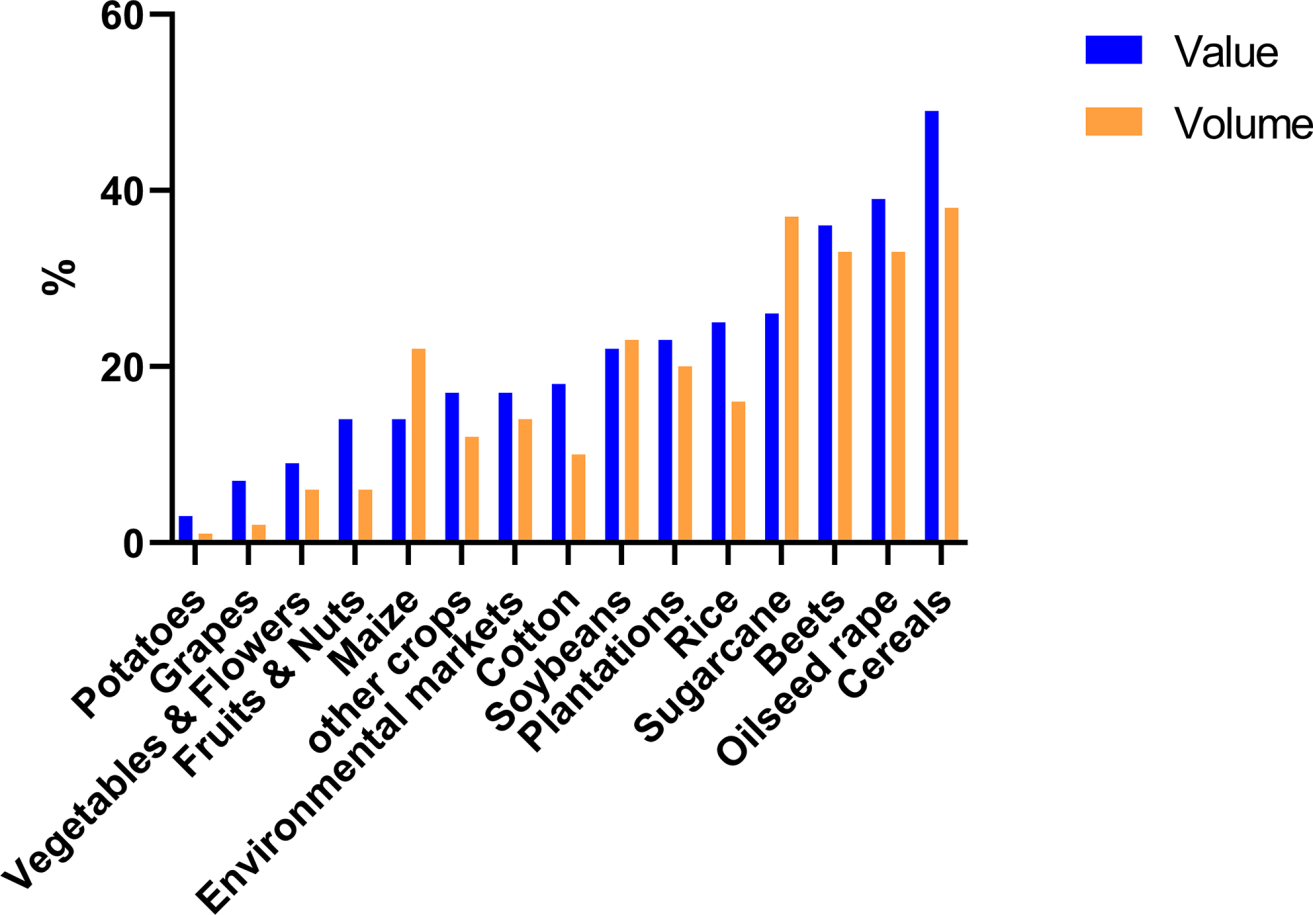
Azoles share of the total fungicide market (%) measured as both volume and market value in different crop segments. Data from 2019 (source: Bayer Internal Source).

The total market of DMI fungicides has been consistently large and significant for many years (Bayer Internal Source and AgbioInvestor/Kynetec). The volume of DMIs has doubled over the last 25 years, and the market value has increased more than four times (**Figure 1**). The DMIs currently have about a 16 % share of the global fungicide volume market, steadily increasing since the 1990s (**Figure 2**) (Bayer Internal Source). On the other hand, the value has remained relatively steady, fluctuating between 20 and 25 % of the total fungicide value since the 1990s. This reflects a price drop probably linked to many of the azoles no longer being covered by patents and the generic products subsequently providing a more competitive environment. The largest volumes of azoles are sold in Europe and Asia, involving more than two-thirds of all azoles worldwide (**Figure 3**) (AgbioInvestor/Kynetec). As data indicate, their use in parts of the world with more extensive and lower-yielding arable crop production, such as in Northern America or Australia, is considerably lower. Despite an overall lower use in Northern America the use of azoles has increased more than 4-fold from 2006 to 2016, particularly in wheat, soybean, and corn (Toda et al., 2021). The azoles play a significant role in disease management in all major crops (**Figure 4**).

In 2019 approximately 40 % of the volume of fungicides used in cereals are azoles, and the marked values cover almost 50 %. Azoles play a similar major role in a long list of crops including oilseed rape, sugar beet, soybean, rice, plantations and sugarcane typically covering about 20-30% of the volume used in the specific crops (**Figure 4**). On the crop basis, azoles are less important in potatoes, wine, fruit, nut, vegetable and flower production *versus* arable crops (Bayer Internal Source).

## Use of Azoles

### Seed Treatment

One of the greatest success stories in plant disease management represents the use of fungicides as a seed treatment. A very high proportion of cereal seed used for drilling is treated with effective treatments to avoid possible problems with seed-borne diseases. Typical treatment targets include smut and bunt diseases (e.g. *Ustilago* spp., *Tilletia* spp.), Fusarium diseases (e.g. *Fusarium* spp., *Microdochium* spp.) and leaf blotch diseases (*Pyrenophora graminearum/teres*) in barley, *Parastagonospora nodorum* in wheat) (Lamichhane et al., 2020).

The risk of infections of wheat with common bunt (*Tilletia caries*) has been recognized since ancient times occasionally causing major losses in yield and quality (Fischer and Holton, 1957). Since the 1970s, azoles (e.g. tebuconazole) have dominated and replaced unwanted products like the organic mercury fungicides (Eastmond and Balakrishnan, 2010), which had a detrimental environmental impact. Also, the Fusarium/Microdochium complex has traditionally been controlled by seed dressings containing azoles (e.g. bitertanol, difenoconazole, triticonazole, prothioconazole and tebuconazole) (Glynn et al., 2008). The risk of seedling blight is potentially high following seasons with particularly wet conditions during flowering and ripening (Osborne and Stein, 2007).

Seed treatments are generally considered less environmentally harmful because they are applied directly to the seed at a significantly reduced application rate compared with foliar applications (Lamichhane et al., 2020). Although thresholds for the application of seed treatments have been proposed (Cockerell et al., 2001; Nielsen, 2003), in many countries, most seed lots are still treated regardless of the infection levels of the seeds, partly due to the short interval between harvest and sowing.

### Small Grain Cereals

Azoles are widely used in cereal crops, such as wheat and barley. More than one third of all fungicides applied in cereals globally are azoles (AgbioInvestor/Kynetec). Fungicide use in cereal crops has traditionally been highest in Europe. More specifically, fungicides are especially frequently used to protect wheat crops (Kuck and Gisi, 2006). Wheat production in Europe represents a very intensive cropping system and cereals are commonly attacked by the leaf diseases STB and rust diseases caused by *Puccinia striiformis* or *Puccinia triticina*. During heading wheat can also be infected with Fusarium head blight (FHB) caused by various species of Fusarium. Attack of Fusarium can cause a development of undesirable content of mycotoxins, which reduce the quality and feeding value of the grain (Bottalico and Perrone, 2002).

Winter wheat crops mostly receive between one and four fungicide treatments per season, depending on the climate and regional differences (Jørgensen et al., 2014; Turner et al., 2021). Fungicide use in other cereals, such as barley, rye, and oat crops, is less frequent; up to twice per season (Jalli et al., 2020). Fungicide sprays aim to protect the upper leaf layers and the ear, contributing most to retaining yields (AHDB, 2021b). The spraying intensity in Europe is highly correlated to humidity events in the cropping season. In countries with a lot of rainfall, such as the Great Britain and Ireland, fungicide treatments carried out at the right time typically result in a yield benefit of around 2 t/ha, increasing the yield up to 20 % (Fones and Gurr, 2015). In areas with fewer humidity events and fewer treatments required, yield responses from fungicide treatments are commonly lower; in the range of 0.5 to 1.0 t/ha (Jørgensen et al., 2014). Data from the England and Wales show that the number of fungicide treatments per season since 2014 has exceeded 3.5 times on average, and azoles are used on all farms and are included in more than 80 % of the treatments carried out as T1 (growth stage (GS) 32-33 BBCH), T2 (GS 39) and T3 (GS 59-65) (Turner et al., 2021). Data from Germany and Denmark have also shown a widespread use of azoles in winter wheat (Lamichhane et al., 2016; Heick et al., 2020a). Regarding control of FHB, azoles (e.g tebuconazole, metconazole and prothioconazole) are currently the only group of fungicides, which offers significant reductions in both severity and the level of mycotoxins in grain (Edwards and Godley, 2010).

The importance of azoles for cereal production was highlighted by Blake et al. (2011), who investigated the impact of a partial or complete azole ban in cereals in three European countries (Great Britain, France and Denmark): The value of azoles to wheat production was estimated at € 1.071 million per year, and an estimated reduction in wheat production was found in the range of 5 to 9 %. This reduction was linked mainly to the lack of control of STB in the absence of azoles in the commonly used fungicide applications.

Current developments in fungicide registration in Europe hint at a paradigm change, significantly impacting cereal production. The European growers are facing a reduced availability of fungicide groups for control of leaf diseases in cereals (Bryson and Brix, 2019). The main uses currently include the azoles (e.g. prothioconazole, epoxiconazole, tebuconazole, mefentrifluconazole), succinate dehydrogenase inhibitors (SDHIs) (e.g. bixafen, fluxapyroxad, fluopyram), quinone outside inhibitors (QoIs) (e.g. pyraclostobin, azoxystrobin) and the multi-sites, such as folpet and sulfur. The multi-site inhibitor, chlorothalonil, that previously was most widely used has been prohibited as a control option in the EU since 2020, leaving a significant gap in adding effective multi-sites as part of an anti-resistant strategy.

Disease management is challenged as a result of problems with fungicide resistance in populations of, for example, *Zymoseptoria tritici* (STB)*, Pyrenophora teres* (net blotch) and *Ramularia collo-cygni* (Ramularia leaf spot). The three plant pathogens mentioned above have developed resistance to the major MoA (QoIs, azoles and SDHIs) (Cools and Fraaije, 2013; Dooley et al., 2016; Rehfus et al., 2016; Blake et al., 2018; Rehfus et al., 2019). The QoI group is no longer effective against STB, Ramularia leaf spot and powdery mildew (*Blumeria graminis*).

It is clearly documented that the efficacy of azoles used for the control of *Z. tritici* has been declining in recent years (Cools and Fraaije, 2013; Blake et al., 2018; Heick et al., 2020a; Jørgensen et al., 2021a). The patterns of decreasing field performances have been confirmed by rising EC_50_ values for several azoles (Blake et al., 2018; Huf et al., 2018). The level of resistance is found to be highly influenced by the local risk of STB, the strategy of fungicide use and the level of intensity in the control program (Heick et al., 2017; Jørgensen et al., 2021a).

Although resistance to azoles is widespread in *Z. tritici*, a recent investigation has shown that the efficacy of individual azoles varies significantly across Europe, with reduced effects going from east to west (Jørgensen et al., 2021a). Nevertheless, epoxiconazole, prothioconazole, tebuconazole, and metconazole gave an average of 55 to 65 % control, indicating a very similar control overall against STB. Despite the relatively high level of azole resistance, the applications of azoles still resulted in approximately 10% yield increases (Jørgensen et al., 2021a). A new azole, mefentrifluconazole, was introduced into the cereal fungicide market in 2020. It is assumed that mefentrifluconazole will supplement and partially replace the older azoles due to its high intrinsic activity particularly on STB, as shown in several trials across Europe (Jørgensen et al., 2020a).

### Oilseed Rape

Azoles are frequently applied to control the major diseases in oilseed rape (**Figure 4**). Oilseed rape is used both for the production of edible oil and biodiesel. The worldwide area grown with oilseed rape (incl. canola) has increased about 50 % from 1995 to 2019 (**Table 3**) and the production has increased by about 80 % during the same period. Consequently, the disease pressure of major oilseed rape diseases has also increased. Several different fungicide classes have proved effective globally for the control of serious diseases in oilseed rape including DMIs (Li et al., 2015), QoIs (Xu et al., 2014) and SDHIs (Stammler et al., 2007). Approximately 5 % of all azoles used are applied in oilseed rape (AgbioInvestor/Kynetec).

**Table 3:** Data illustrating the intensification of agricultural production during the last 25 years (FAOstat).

	Area M ha		% area increase	M tons produced		% increase	Yield per ha		% increase
	1995	2019		1995	2019		1995	2019	
Wheat	216	216	0	544	766	41	2.52	3.55	41
Barley	68	51	-25	140	158	13	2.06	3.10	50
Rice	150	162	8	547	755	38	3.65	4.66	28
Soybean	62	120	94	126	336	167	2.03	2.80	38
Sugar cane	18	16	-11	1163	1949	68	64.61	121.81	89
Oil seed rape	23	34	48	39	70	80	1.70	2.06	21
Sugar beet	7,7	4,6	-40	264	278	5	34.29	60.43	76
Banana	3,8	5,1	34	58	116	100	15.26	22.75	49
Maize	135	197	45	517	1148	122	3.83	5.83	52

Azoles are commonly applied in the fall in the maritime zone of Europe to control diseases such as light leaf spot *(Pyrenopeziza brassicae*) and canker/Phoma (*Plenodomus lingam, P. biglobosa*) and for their growth-regulating properties (Berry and Spink, 2009). In the absence of fungicides, severe attacks of light leaf spot can lead to yield losses of up to 1 ton/ha (AHDB, 2021a), and yield losses due to Phoma may be up to 0.5 ton/ha in susceptible varieties. Oilseed rape is at risk of developing severe attacks of Sclerotinia stem rot (*Sclerotinia sclerotiorum*) in the spring (Derbyshire and Denton‐Giles, 2016). Oilseed rape is commonly sprayed once or twice per season in major parts of Europe to prevent these diseases using azoles alone or in a mixture with a QoI or a SDHI. The azoles most commonly applied are prothioconazole, tebuconazole, and difenoconazole. It is difficult to predict the risk of attack by Sclerotinia stem rot, and an element of insurance treatment is common to remove the risk of unpredictable attacks (Derbyshire and Denton‐Giles, 2016). An annual loss in the Great Britain due to diseases has been estimated to about 5 % for an average crop yield of ca. 3 ton/ha (AHDB, 2021a). Apart from azole resistance in the population of *Pyrenopeziza brassicae* (Carter et al., 2014), there have not been clear reports of fungicide resistance to azoles in oilseed rape diseases.

### Sugar Beet

Sugar beet is a vital crop grown primarily in the temperate region for sugar production. The worldwide cropping area covers approximately 4.5 million ha, with roughly 70 % sugar beet production in Europe and large production in the US (FAOstat). Azoles are used mainly against foliar diseases, such as powdery mildew (*Erysiphe betae*), beet rust (*Uromyces betae*), Cercospora leaf spot (*Cercospora beticola*) and Ramularia leaf spot (*Ramularia beticola*). The effective control of foliar diseases is an essential factor for securing yields and exploiting the crop’s full yield potential. Yield gains from fungicides in the range of 10 to 20 % are not uncommon following effective treatments against mildew and rust (Elliott and Weston, 1993; Stevens and Burks, 2012; Heick et al., 2020b). Cercospora leaf spot is the most important disease in continental Europe, and yield losses of 50 to 70 % are possible if not controlled (Rangel et al., 2020). Tetraconazole, difenoconazole, prothioconazole, epoxiconazole, propiconazole, and cyproconazole are among the common azoles used alone or in combination with QoIs, SDHIs or multi-site inhibitors. Major problems with fungicide resistance are seen, particularly regarding the control of Cercospora leaf spot, due to intensive spraying in parts of Europe and the US (Bolton et al., 2013; Muellender et al., 2021).

### Soybeans

Soybeans are an important protein crop cultivated throughout the world. The primary production occurs in Asia and the Americas, where cropping systems rely on high fungicide inputs to achieve a high yield. The area grown with soybeans has almost doubled during the last 25 years, and the acreage has increased dramatically, particularly in South America (**Table 3**).

Several fungal pathogens may attack the crop during the season and lead to severe yield losses. The most important disease is Asian soybean rust (SBR) caused by *Phakopsora pachyrhizi*. The use of fungicides has increased significantly in this crop from the beginning of this century in Latin America after the introduction of SBR to this continent (Ivancovich, 2005; Schneider et al., 2005). The SBR can reduce the attainable yield by up to 78 % during severe epidemics (Dalla Lana et al., 2015). The environmental conditions in countries such as Brazil are often favorable for the development of SBR during the cropping season (Del Ponte and Esker, 2008; Li et al., 2010), where inoculum of *P. pachyrhizi* can have a year-round survival. Resistant varieties are not yet commercially available, and frequent fungicide applications are considered the effective tool to protect yield. Several azoles have shown good control against the disease (Schmitz et al., 2014).

Other important pathogens in soybean are *Cercospora kikuchii* causing Cercospora leaf blight and purple seed stain and target spot (*Corynespora cassiicola*). Yield suppression caused by target spot was estimated at 20 to 40 % in the United States (Koenning et al., 2006). Significant control of the three main diseases was obtained from Argentina using mixtures of azole, SDHI and QoI fungicides (Reznikov et al., 2019). Approximately 15 % of all azoles sold globally are used in soybean (AgbioInvestor/Kynetec).

Following the intensive use of azoles over the years, fungicide resistance has been reported in *P. pachyrhizi* populations with decreased sensitivity to azoles (Schmitz et al., 2014), as a consequence reduction in DMI efficacy to *P. pachyrhizi* has been reported from, for example, Brazil (Scherm et al., 2009; Reis et al., 2015). As also seen for other fungi the insensitivity to DMIs is associated with point mutations in the *cyp51* gene or *cyp51* overexpression (Schmitz et al., 2014; Dalla Lana et al., 2018). General recommendations for using azoles only in mixtures and introducing a crop-free period resulted from a shift in azole sensitivity in the *P. pachyrhizi* population in recent years.

### Banana

Bananas are one of the most important crops and rank fourth among crops that supply carbohydrates for humans and provide food security for millions of people (Jones, 2000). The global banana production is big, producing more than 100 M tons (**Table 3**). About 85 % of the banana export for the US, European and Japanese markets are from Latin America (Pocasangre et al., 2017).

Banana plantations are particularly susceptible for diseases due the cultivation in monocultures. Among the banana diseases, black sigatoka caused by *Pseudocercospora fijiensis* is the most damaging foliar disease in banana production and has clearly a higher economic importance compared to yellow sigatoka, caused by *Pseudocercospora musae* (Mehl and Manger-Jacob, 2015). It causes severe leaf defoliation and indirect post-harvest fruit quality problems due to premature ripening of the fruit, making it unacceptable for export (Ploetz, 2000; Marín et al., 2003). Other important diseases are Panama disease caused by *Fusarium oxysporum* f. sp. *cubense* (FOC) and anthracnose caused by *Colletotrichum musae.*

DMI fungicides are particularly of high relevance for the management of black sigatoka in banana. Dependent on the country and banana growing region, the number of total fungicide treatments and DMI applications within spray regimes vary significantly. In a global survey for DMI sensitivity of around 600 isolates collected between 2011 and 2014 in Cameroon, Colombia, Costa Rica, Dominican Republic, Ecuador, Guadalupe, Martinique, and the Philippines, the number of DMI cycles was estimated from, e.g., 7 out of 56 in Costa Rica, 13 out of 30 in Ecuador, or 9 from a total of 11 cycles in Martinique (Chong et al., 2021). This varying and too intensive use of DMIs has led to DMI shifting over the baseline sensitivity.

Decreased DMI sensitivity has been described to be based on several point mutations in the *cyp51* gene of *P*. *fijiensis* (Cañas-Gutiérrez et al., 2009), and on overexpression of the *cyp51* gene (Diaz-Trujillo et al., 2018). Latest details on the understanding of DMI resistance mechanisms in *P*. *fijiensis* field populations have been recently summarized (Chong et al., 2021), and an apparent link has been seen between increasing EC_50_ values and the increasing amounts of fungicides applied.

To further limit sensitivity shifting and to avoid overuse, for black sigatoka control in banana, DMI fungicides are by FRAC (www.frac.info) recommended to be exclusively applied in mixtures and in full alternation with other, non-cross resistant modes of action. In addition, a maximum of eight applications containing DMI fungicides is recommended and DMI’s must not be included in more than 50 % of the total number of sprays.

As a result of the very frequent fungicide applications including various mode of actions which are considered to have an extremely high environmental and economic burden (Risède et al., 2010), the demand for organically grown bananas has increased significantly and is now a widespread segment in parts of Europe. Organic growers replace classical fungicides mainly with petroleum oils, which have been used for control since around 1950 (Guyot and Cuillé, 1954), but yields are often reduced to about half the yield of conventional ones as a result of the oils being less efficient and also impacting photosynthesis yields in organic plantation (Pocasangre et al., 2017).

### Rice

Rice is divided in two species: Asian rice or, less commonly, African rice. Rice is among the most important food crops, feeding more than 50 % of the global population (Liu et al., 2007), particularly in Asia and Africa. Various phytopathogenic fungi cause significant attacks in rice. Among the most serious are leaf and panicle blast (*Pyricularia oryzae*), which is considered the most widespread and yield-reducing disease in rice, attacking both lowland and upland rice. Yield reduction in the range of 10 to 30 % is commonly reported (Dean et al., 2012). Sheath blight (*Rhizoctonia solani*), brown spot (*Cochliobolus miyabeanus*) and false smut (*Villosiclava virens*) are also important diseases in rice (Deepan et al., 2017). Host resistance is important when managing diseases in rice, for example, rice blast; however, many resistant cultivars released have a short life because of the highly diverse genetic structure and quick evolution of particularly *P. oryzae* that overcomes resistance (Skamnioti and Gurr, 2009). Consequently, fungicide application plays an important role in rice blast management. Several groups of fungicides are used to control the main diseases in rice. Azoles, which include propiconazole, tebuconazole, and epoxiconazole, are commonly used for the control of both seed-borne and leaf diseases in rice. Approximately 10 % of all azoles sold globally are used in rice (AgbioInvestor/Kynetec).

Several issues with fungicide resistance in the population of *P. oryzae* have been identified (Kim et al., 2003; Zhang et al., 2009), but so far, only minor problems with resistance are reported from azoles (Yan et al., 2011).

### Citrus

Citrus fruits are among the ten most important crops in terms of total fruit yield worldwide and rank first in the international fruit trade in terms of value. More than seven million hectares are planted with citrus throughout the world, including oranges, lemons, grapefruits, pomelos, and limes. Pathogenic fungi can attack plants of the citrus genus during the cropping season, causing yield losses or external blemishes and reducing fruit size, but can also cause tremendous post-harvest loss (Sonkar et al., 2008). Fungicides, including azoles, are commonly applied at times to both secure yield and market value and reduce post-harvest losses. The most important fungal diseases that attack citrus trees are dieback/mal secco of citrus (*Plenodomus tracheiphilus*), postbloom fruit drop (*Colletotrichum* spp.), Alternaria brown spot (*Alternaria* spp.), citrus scab (*Elsinoe fawcettii*), greasy spot (*Zasmidium citri-griseum*) and melanoses of citrus (*Diaporthe citri*) (Timmer et al., 2004; Khanchouch et al., 2017).

Citrus spp. are frequently sprayed during the season, commonly in seven- to twenty-one-day spray intervals, depending on the product and disease severity, to diminish the risk of crop losses. Different azoles are registered for the control of the diseases mentioned above depending on the growing region. The azoles most commonly used are difenoconazole, fenbuconazole, imazalil, mefentrifluconazole, and propiconazole (https://edis.ifas.ufl.edu/pdffiles/CG/CG10100.pdf). Azoles are often applied in mixtures with QoIs or SDHIs (Silva-Junior et al., 2014).

The most harmful post-harvest phytopathogenic fungi of oranges are *Penicillium digitatum*, which causes the green mold disease responsible for about 90 % of post-harvest losses (Costa et al., 2019; Papoutsis et al., 2019), and *Penicillium italicum*, the agent causing blue mold disease.

The control of post-harvest rots in citrus relies on cool storage combined with the application of coatings (e.g. wax) containing fungicides, such as imazalil or pyrimethanil (Sonkar et al., 2008). Fungicides are often applied when fruits are cleaned, brushed and waxed. Fungal resistance to these chemicals, along with consumer pressure for safer control methods, is providing the impetus for different treatments based on alternatives in combination with heat treatment and biological control agents, such as naturally occurring bacteria and yeasts.

### Amenity Grass

Fungicide application on amenity areas does not impact food production or secure crop quality but aims to provide optimal conditions for e.g. sports activities like golf. Golf sport is played around the globe with a total number of golf courses reaching nearly 39.000 by end 2018, spread across 209 of the world’s 249 countries (Anon, 2019).

Different species of turfgrass on golf courses under intensive management are often subject to outbreaks of infectious diseases. Cultural conditions that predispose turfgrass to diseases include close mowing, inadequate or excessive nitrogen fertility, sparse or frequent irrigation, excessive thatch, poor drainage and shade (Clarke et al., 2020).

Diseases might cause excessive damage to highly managed turfgrasses even when good turfgrass management practices are followed. The list of possible attackers is long and includes dollar spot (*Clarireedia jacksonii and C. monteithiana*), Anthracnose (*Colletotrichum cerale*), brown and large leaf patch (*Rhizoctonia solani*), red thread (*Laetisaria fuciformis)*, pink snowmold (*Microdochium nivale*), summer patch (*Magnaporthiopsis poae*), necrotic ring spot (caused by *Ophiosphaerella korrae*), take-all patch (caused by *Gaeumannomyces graminis*) and spring dead spot (caused by *Ophiosphaerella herpotricha*). The DMI fungicides are known to be effective against many of the mentioned diseases. Several azoles, including tebuconazole, fenarimol, triticonazole, metconazole, propiconazole, myclobutanil, triadimefon, and mefentrifluconazole, are recommended for use on golf courses in, for example, the US. Mainly mixtures including actives with different MoA (SDHIs, QoIs, multi-site inhibitors) are recommended due to significant problems with fungicide resistance (Clarke et al., 2020).

### Azoles as Growth and Stress Regulators

Certain azole compounds interfere with the biosynthesis of gibberellins, plant hormones that regulate various developmental processes, including stem elongation, germination, dormancy, flowering, flower development, and leaf and fruit senescence. As a result, several azole derivatives have been developed as growth regulators and are recommended worldwide for plant growth regulation (Fletcher et al., 2010). Paclobutrazol, uniconazole, and metconazole are among the azoles most widely used for growth regulation. The morphological changes induced by azoles include reducing plant height, a higher root-to-shoot ratio and modified leaf morphology. The application of azoles is a standard practice to manipulate the shape, size, form and esthetic quality and extend the marketing period of many ornamental plants (Fletcher et al., 2010). The production of pot plants in countries such as the Netherlands and Denmark is significant, and the market value is linked to manipulating growth. The application of plant growth regulators is also a standard practice for many bedding plants to maintain quality and compactness before a sale and increase post-transplant survival (Davis et al., 1991; Keever and Foster, 1991).

Azoles have also helped to control the growth, lodging resistance, and cold hardiness of some important crops such as oilseed rape and tomato (Fletcher et al., 2010), and likewise azoles have shown potential use in regulating the growth and yield of several fruit and nut species, including pome fruits, stone fruits, nut species, and some tropical and subtropical fruits (Gaash and David, 1989; Stan et al., 1989; Tukey, 1989).

### Wood Preservation

Azoles are commonly used as wood preservatives against a wide range of wood-destroying fungi. Their use aims to ensure the long-term stability of wood products. Azoles were first introduced to the wood preservation market in the 1990s and are now among the fungicides most commonly used in the wood industry (Derkyi, 2020). Tebuconazole accounts for the largest share of azole demand across all industries globally, with 16,000 tons consumed per year and a market of $ 600 million in 2015 (Kukowski, 2018). Azoles are estimated to cover about 18 % of the total wood preservation market, with more than 50 % being sold in North America (personal communication, Andreas Goertz, Bayer). Propiconazole, tebuconazole, and cyproconazole have good activity in solvents and water-based formulations against wood-destroying Basidiomycota. The pathogens claimed to be controlled include *Lenzites trabea, Rhodonia placenta, Trametes versicolor,* and *Coniophora puteana* (Bruns et al., 2005). The benefits of azoles are that they are UV-stable and have good stability in treated wood, thus, they are suitable for long-lasting protection of wood against decay fungi (Goodwine, 1990; Buschhaus, 1992). Most commercial wood preservation treatments contain copper ions, which give treated wood its characteristic greenish-brown coloration. Several preparations, which typically include copper have been commercialized and are composed of 96 % ammine copper and a 4 % azole, either tebuconazole used alone or a mixture of propiconazole and tebuconazole (Derkyi, 2020).

## Azole Use in a Greater Context

### Overall Benefits

As described above azoles have been a key fungicide group for the control of major diseases in a vast range of crops for more than 40 years. They are applied as either seed treatment, foliar application, or post-harvest treatment. A significant intensification of arable cropping has taken place during the same period, which has led to significant increases in the arable area cropped and production units per ha. The increase in the total area over the last 25 years has been most pronounced for soybean, corn, oilseed rape, and bananas, while the increase in yield per ha has been most pronounced in sugar beet, corn, bananas, wheat, and barley (**Table 3**). These increases can partly be attributed to the intensification of breeding efforts and the general improvement of cropping systems. Also, the use of plant protection products has played a significant role in the increased yields per ha. Schreinemachers and Tipraqsa (2012) calculated that a 1.8 % increase of pesticide input per ha led to a 1 % increase in crop output per ha. Linked to the big increase in yields per ha, the global increase in total pesticide sales has increased most in Europe, Asia, and Latin America, as shown in Lamichhane et al. (2016).

Azoles with a high efficacy already at low rates and often with clear visual benefits have been rapidly adopted by growers worldwide in many crop production systems. Despite intensive use and in contrast to other systemic and target-site fungicides, the azoles are still contributing positively to a wide range of disease control, as can be seen, for example, in winter wheat (Jørgensen et al., 2021a). This constant benefit has been driven by the development of more potent azoles over the years (Russell, 2005; Parker et al., 2011; Tesh et al., 2019) and the moderate risk of resistance development (www.FRAC.info). In practice, azole compounds have been widely used either as solo products or in a mixture with other active ingredients to broaden the control spectrum or minimize the risk of resistance development.

Despite many positive benefits reported from the use of azoles across many different crops and sectors, this group of fungicides has been under increasing public pressure in recent years due to their intensive and increasing use in general and their potential impact on the environment and health risks specifically (Taxvig et al., 2007; Verweij et al., 2016; Rosenbom et al., 2020).

### Risk of Resistance in Plant Pathogens

Despite DMI fungicides having been at the forefront of the control of fungal plant pathogens for over 40 years and being challenged by resistance issues (Brent and Hollomon, 2007), their use has increased significantly during the last 25 years (**Figure 1**). Azole resistance has been reported in 30 plant pathogens (www.frac.info), in over 60 countries (Fisher et al., 2018). The evolution of resistance has especially been recognized in pathogens that sporulate profusely and have short generation times. Indeed, resistance evolved in the ‘high-risk’ cereal powdery mildews (*Blumeria graminis*) within four years after introducing the azoles triadimefon and triadimenol in the late 1970s (Senior et al., 1995). Despite this, several azoles (e.g. prothiconazole) still provide moderate to good control of powdery mildew, indicating incomplete cross-resistance (Tucker et al., 2019). Subsequently, problems with shifting sensitivity or resistance took place in many other plant pathogens and are particularly well-documented for *Z. tritici* (Leroux et al., 2007; Cools and Fraaije, 2013; Blake et al., 2018; Jørgensen et al., 2021a).

The development of resistance is of major concern for economically important diseases for which azoles have been used widely – either as solo or as mixing partners. In several cases, it has been challenging to replace the azoles with other similarly effective fungicides. The increasing problems with resistance have necessitated recommending anti-resistance strategies using alternation or mixtures of active compounds with different MoA, applying fewer treatments and reducing the dose to minimize selection pressure (van den Bosch et al., 2014). Successful mixing normally includes actives from different MoA groups. However, due to incomplete cross-resistance benefits from mixing azoles have been shown where isolates, with specific mutation conferring higher levels of resistance against some compounds and lesser resistance to others (Kildea et al., 2019; Heick et al., 2020a). For e.g. *Z. tritici*, isolates which are highly resistant to tebuconazole but fully susceptible to, for example, prochloraz has been recognized (Leroux et al., 2007). It is currently common practice to mix azoles, which have shown different degrees of incomplete cross-resistance (Jørgensen et al., 2018).

### Integrated Pest Management

Integrated pest management is a concept much promoted when politicians, administrators, advisors, and farmers talk about crop protection (Lamichhane et al., 2016; Vasileiadis et al., 2017). The approach includes elements such as using resistant cultivars, a threshold concept ahead of pest control measures, and an overall reduction in the amount/frequency of pesticides. The general aim is that pesticides should be applied at an economically and ecologically acceptable level, as stated in the EU directive 2009/128/EC, aiming at achieving the sustainable use of pesticides. Negligible crop losses due to pests are economically acceptable. However, an increase in crop productivity without adequate crop protection does not make sense because an increase in attainable yields is commonly associated with an increased vulnerability to damage inflicted by pests and diseases (Oerke, 2016). The benefits of using fungicides in e.g. wheat have been investigated in several studies and generally prove cost-effective (Leadbeater et al., 2000; Jørgensen et al., 2014; Fones and Gurr, 2015). It is, however, worth remembering that the economic return from fungicide input is highly variable depending on the specific season. The net yield responses from standard fungicide treatments in Northern-European countries are negative in 1 to 2 seasons out of 10 (Jalli et al., 2020), which indicates that no fungicide treatments should have been carried out in those years. This highlights the necessity of introducing prediction schemes to support farmers’ decision-making (Jørgensen et al., 2020b).

Although chemical control of fungal diseases plays a significant role in reducing crop losses, there is a tendency for several cropping systems to become overly reliant on the use of fungicides; these systems also show tendencies of overusing them. The reason for the excessive use of fungicides is manifold. Groups of farmers are risk-averse and disapprove of the concept of accepting negligible losses and attacks below a certain threshold. This often leads to additional, unnecessary fungicide applications or the use of higher doses as a certain ‘element of insurance’ (Hardwick et al., 2001; Te Beest et al., 2013). This use pattern based on an insurance principle is partly due to a lack of or inadequate and ineffective risk-predicting tools of epidemic developments of plant pathogens (Jørgensen et al., 2017). Furthermore, the advisors’ background influences the input recommended, where independent advisors have been found to recommend lower input than supplier-affiliated ones (Pedersen et al., 2019).

Alternative methods of disease control are available for a significant number of plant pathogens. Varietal resistance is seen as the most important element when wishing to minimize diseases and losses due to plant pathogens (Singh et al., 2016; Pocasangre et al., 2017). Unfortunately, resistant varieties do not solve all problems, as resistant cultivars are not available in all crops and not stable. Many situations occur where pathogens have overcome the resistance in a specific crop (Skamnioti and Gurr, 2009; Singh et al., 2016; Vagndorf et al., 2019), which leads to the fact that fungicides are likely also to play a significant role as a tool for minimizing losses in high-risk situations in the future.

Along with the increasing concerns over toxicological and environmental issues regarding pesticides, an increasing interest in biological control agents has led to a search for potential candidates to replace or supplement synthetic fungicides, intending to move in the direction of more sustainable agriculture (Wei et al., 2016). The hope is that these substances, for example, different *Bacillus* spp., can replace traditional chemistry – a task that is proving quite difficult (Kildea et al., 2008; Reiss and Jørgensen, 2017; Matzen et al., 2019; Dadrasnia et al., 2020). Alternative methods of disease control for a significant number of plant pathogens are, by and large, either unavailable or have so far proven difficult to develop.

### Azoles and Their Metabolites in Water

Investigations have demonstrated that frequently used azole compounds, pharmaceuticals as well as pesticides/biocides, are continuously released and are widespread in the aquatic environment following direct or indirect discharge of wastewaters (Kahle et al., 2008; Chen and Ying, 2015). The residues of azole fungicides could cause toxic effects on aquatic organisms such as algae and fish.

The substance 1,2,4-triazole is a metabolite derived from several widely used azole fungicides. This substance has recently been detected in groundwater and drinking water samples undergoing extended investigations in Danish surveys (Rosenbom et al., 2020). These findings have led to the Danish Environmental Protection Agency enforcing restrictions in 2014 on certain azole fungicides (Rosenbom et al., 2020). However, not all cases of findings of 1,2,4-triazoles can be clearly linked to the use of azoles, which indicates a background level of unknown origin of 1,2,4-triazole in the soil environment. The use of azole fungicides for wood preservation and the use of 1,2,4-triazole as a nitrification inhibitor locally could have some influence on the levels measured (Cowi, 2019). Finally, it cannot be excluded that 1,2,4-triazole is also formed by natural processes in the environment, including some natural strains of microorganisms (Blank et al., 2018; Cowi, 2019).

### Impact on Human Health

Two significant issues have been increasingly discussed concerning the health aspects of authorized azoles. One element is their potential activity as endocrine disruptors (Taxvig et al., 2007), the other is a selectivity factor for resistance to *Aspergillus* fungi, potentially impacting human health (Verweij et al., 2016).

#### Endocrine-Disrupting Features

The mode of action of azole compounds implies a potential to affect the endocrine systems of different organisms (Taxvig et al., 2007). Criteria for assessing endocrine-disrupting properties have been established under the EU pesticide regulation (Anon, 2017a), which is also expected to impact the future authorization of azole fungicides. Azole inhibition of P450 cytochromes is not specific to CYP51; other sterol enzymes, including aromatase (CYP19), the enzyme responsible for converting androgens to estrogens, can also be affected (Zarn et al., 2003). Endocrine-disrupting chemicals are seen to affect the organism at very low doses and are found especially harmful if the exposure occurs at critical times in the body’s development, including prenatal and pubertal development. It has been shown that azole fungicides differ widely in their potency to inhibit the human aromatase enzyme (Trösken et al., 2004). Consequently, the screening strategy for new azole fungicides aims to identify candidates with a high fungicidal activity and minimal likelihood of adverse side effects that would indicate an endocrine-disrupting potential (Tesh et al., 2019).

#### Aspergillus

*Aspergillus fumigatus* is a mold commonly found in soil and decaying organic matter (Bignell, 2014). It is also an opportunistic human pathogen causing allergic symptoms and life-threatening invasive infections (aspergillosis) in patients with immunodeficiency or who are immunocompromised. The incidence of invasive aspergillosis has been increasing in recent years following an increased number of immunocompromised individuals in the population. Only a few effective antifungal drugs are available for the clinical treatment of the disease. Among those, azoles (voriconazole, isavuconazole, posaconazole, and itraconazole) play an important role. Cross-resistance has been verified between clinical azoles and agricultural azoles (Snelders et al., 2012). Highly azole-resistant isolates have been found in the Netherlands in azole-naïve patients since 2007 and are now recognized globally (Verweij et al., 2016). As a result, the evolution and spread of pan-azole-resistant alleles in clinical and environmental isolates of *A. fumigatus* is increasingly regarded as a global human health concern (Chowdhary et al., 2013; Verweij et al., 2020).

*A. fumigatus* shares the natural environment with plant pathogens and is also exposed to selective pressure from azole fungicides when these are applied for control of plant pathogens. The emergence of resistant *A. fumigatus* in fields in several studies was closely related to residual levels of azole fungicides, and composting environments have particularly proved to impact development (Anon, 2017b). An extensive Chinese survey has linked the prevalence of resistant *A. fumigatus* in agricultural fields to the applications of azole fungicides (Cao et al., 2021). Specific studies showed variations in the different azoles’ selection potential as a result of variable sensitivity and affinity to the target gene in *A. fumigatus* (Snelders et al., 2012; Jørgensen et al., 2021b). Although a lot of research has been dedicated to this topic in recent years, the role of azole fungicides for resistance development and occurrence in *A. fumigatus* has not yet been fully elucidated (Gisi, 2014; Barber et al., 2020; Fraaije et al., 2020).

### Constraints Due to Stricter Rules for Authorization

The cost and difficulty of discovering and registering new fungicidal compounds have led to a declining product pipeline of new fungicides (Bryson and Brix, 2019). This lower rate and the increasingly adverse regulatory environment, especially in Europe, have resulted in the withdrawal of many current actives, including several azoles (e.g. propiconazole, bitertanol). Several of the azoles have also been categorized as candidates for substitution, which was introduced as part of EU registration in 2009 (Reg EC 1107/2009). It has been estimated that approximately 70 % of the fungicide products registered in the EU contain an active ingredient, which will be prohibited due to cutoff criteria or be listed as a candidate for substitution (Bryson and Brix, 2019); among those listed are epoxiconazole, tebuconazole, difenoconazole, and metconazole. If all these compounds were lost, it could have a significant impact on the arsenal of fungicides and, thus, the options for disease control. The high cost of developing new plant protection products, including azoles, estimated lately to be in the range of $ 300 million, will probably keep the number of new azole candidates down (Bryson and Brix, 2019).

### Public Acceptance

Along with the constraints from the stricter regulatory system, as seen in the EU, increasing concerns regarding the use of pesticides, in general, have been raised by policy-makers and consumers. The market for organically grown products has increased, especially in Western cultures. This demand is increasingly met by retailers who support or even drive this development. This trend impacts on fruit and vegetable production today and will probably also impact major arable crops in the future. The size of this trend is difficult to predict because the impact on global food production is very uncertain and major global cultural differences will add to the uncertainty of this process. The increased awareness of pesticides’ environmental fate will undoubtedly fuel the debate on agriculture’s use of pesticides. Concurrently, with more precise detection methods and more thorough screening for pesticide residues, it is crucial to raise the question of acceptable limits and their public acceptance.

In the future, the development of azoles that are not structurally related to medical azoles should have preferences in agriculture. Microbiological methods, screening for MIC_50_ values, molecular biological studies, and protein modeling, are expected to support future azole development to achieve a better specificity for the target enzymes and minimize the risk of cross-resistance (Parker et al., 2014). The skill of designing new molecules, which minimize the risk of undesirable effects, is ongoing, as described for mefentrifluconazole in a recent paper (Tesh et al., 2019).

## Conclusion

Disease control is an everlasting challenge that will require all the resources available to produce affordable, nutritious food in a sustainable way. Fungi pose a constant threat to food production and have proven to be highly adaptable. This threat is steadily growing due to increasing problems with fungicide resistance and emerging diseases that challenge arable production. Azoles have despite resistance issues provided a relatively stable control against many important plant pathogens during decades and are key mixing partners of fungicide groups with a moderate or high risk of resistance development, minimizing the overall risk of resistance development.

Disease control in modern agriculture has developed over decades as a complex system of solutions that contribute to an overall goal of producing high-yielding, quality crops. None of the control solutions developed can stand alone and none was proved indispensable individually. The use of fungicides has developed into an integral part of modern agriculture and will continue to be so. Azoles have played a significant role in effectively managing plant pathogens and minimizing losses caused by plant diseases worldwide for almost half a century. This is unlikely to change within the near future. A significant number of azoles are expected to disappear due to stricter regulations, which aim to ensure human and animal health and protect the environment. Meeting the new requirements applies to already registered and new candidate compounds and will be seen as a benefit of all. Whether azoles remain indispensable depends largely on the industry’s ability to develop new effective azoles or replacements for azoles, ensuring disease control and fulfilling the current and future requirements for registration. This will impact the way in which we are heading.

## Author Contributions

Both authors contributed to the article and approved the submitted version.

## Funding

No specific funding or projects have supported the writing of this review. Support has come from the core funding at Aarhus University.

## Conflict of Interest

Outside this work, we have the following potential conflicts to declare: LJ and TH have over the years received research grants (paid to the institution) from BASF, Bayer, Syngenta, Corteva, Adama, UPL, Globachem.

## Publisher’s Note

All claims expressed in this article are solely those of the authors and do not necessarily represent those of their affiliated organizations, or those of the publisher, the editors and the reviewers. Any product that may be evaluated in this article, or claim that may be made by its manufacturer, is not guaranteed or endorsed by the publisher.
